# Direct discharge from the intensive care unit improved patient flow in a resource-pressured health system

**DOI:** 10.1186/s44158-023-00124-4

**Published:** 2023-10-20

**Authors:** E. O’Riordan, K. Maher, Z. O’Hagan, I. Martin-Loeches

**Affiliations:** 1 Department of Intensive Care Medicine, Multidisciplinary Intensive Care Research Organization (MICRO), St James’ Hospital, Dublin, Ireland; 2 Hospital Clinic, Universitat de Barcelona, IDIBAPS, CIBERES, Barcelona, Spain

**Keywords:** Intensive care, End-of-life, Hospital mortality, Direct discharge, Patient safety, Critical care, Resource management

## Abstract

Critical care practice is constantly evolving. Pressures for bed availability in publicly funded healthcare systems have led to an increase in patients delayed in their discharge from critical care to the wards. This has resulted in more patients discharged directly home (DDH) from the intensive care unit (ICU). However, few formal pathways for DDH exist. We have performed a retrospective audit of the patients discharged home from our unit in the largest tertiary referral hospital in the Republic of Ireland from 2017 to 2022 to investigate their characteristics and the safety of this practice, given the understandable patient safety concerns raised.

**Results** In total, 84 patients have been DDH from our unit between 2017 and 2022 from a total of 4747 patients. The overall rate of DDH increased year on year, and the vast majority of these patients were initially admitted from the emergency department or following elective major surgery. Most patients had an APACHE score of less than 11 points, and the majority were admitted for less than 3 days, with single organ failure. There was a gender divide, as greater than 60% of the patients admitted were male, with a mean age of 44.

**Conclusion** DDH has been an important tool in improving patient flow through the hospital, avoiding unnecessary de-escalation to the ward for a select group of critical care patients. The re-admission rate in the year post-ICU discharge was very low, showing that DDH has not adversely impacted patient safety.

## Background

Due to a continuous progression over the last two decades, the management of critically ill patients has adjusted the model of care. Not long ago, patients were only admitted to an ICU if they needed a full pack of invasive mechanical ventilation, cardiovascular, and even more advanced organ support. In addition, discharge from the ICU always was associated with a link to the ward or a step-down unit until it was clear that the patient was adequately showing good and independent recovery from their disease to go home. Due to changes in the appreciation of ICU, modern intensive care medicine has other models of care, such as patients with predeterminate ceilings of care or even not candidates for some procedures [1]. Additionally, constraints in medical resources in some parts of the world delay the discharge from the ICU to the hospital ward, which delays the admission of very sick individuals to an ICU bed [2]. In some cases, the reason for ICU admission can be linked to the need for an advanced support organ device, such as invasive mechanical ventilation due to the primary reason for non-acute respiratory failure (ARF) due to low GCS to protect the patient’s airway [3]. Anecdotally in our unit, after a short period of time in the ICU, some patients stay longer in the unit awaiting a bed in the hospital ward than the time spent requiring ICU care. This leads to the possibility of discharging patients directly from the ICU in whom physiological derangement was corrected.

In this short report, we outline our experience in the largest teaching acute care hospital in the Republic of Ireland. We aim to determine the discharge features, the type of patients, and the complication rate and need for readmission to determine the feasibility of this process of care.

## Material and methods

### Study design and setting

We performed a retrospective observational study including all patients directly discharged home (DDH) from the ICU in St James’ Hospital, a large tertiary-level adult teaching hospital with 1010 beds including 26 dedicated general ICU beds between January 2017 and July 2022. Our institution is a tertiary referral center for major general and gyne oncological, vascular, and urological surgery and has a significant medical oncology cohort as the bone marrow transplant referral center for Ireland.

### Data collection and analysis

Patients were identified using our critical care databases and further information about their ICU pathway from our critical care electronic patient record IntelliSpace Critical Care and Anaesthesia (ICCA) Philips Healthcare, The Netherlands. Information gathered included gender, age at admission, the primary reason for admission, and APACHE score at ICU admission. Duration of respiratory, dialysis, or vasopressor support was also included. In terms of DDH, we collected data about the length of ICU stay, the reason for DDH and readmission to critical care or ward-level care within 1 year. SPSS version 23 by IBM was used for the statistical analysis. Continuous variables are displayed as means.

## Results

**Fig. 1:**
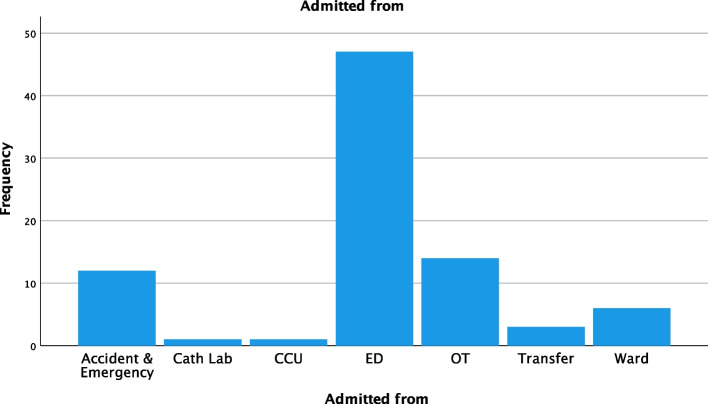
Admission locations for patients directly discharged from the ICU

In total, 84 patients were DDH from the ICU during the period studied, from a total of 4747 patients admitted to our ICU during this time. The overall rate of DDH increased year on year. Between July and December 2017, only two patients were DDH (0.4%) compared to 25 from Jan to July 2022 (5%). Most patients were male (66%), with a mean age of 44. Most patients were initially admitted from the emergency department (70.3%), then the operating room (16.7%), with a minority from other locations (Fig. 1).

The most common primary diagnosis at admission to the ICU was polypharmacy overdose (41.7%). Patients were admitted before DDH 3 days or less (60.7%) with a mean LOS of 4 days and a maximum of 42.

Severity at initial presentation (APACHE II score) was quite varied; however, 60.8% of patients had a score of < 11 with 23.8% having a score of 10/11 which indicates predicted mortality at ICU admission of 15% if non-operative or 7% if postoperative. In general, patients with DDH required little organ support during their stay, in keeping with being generally well and admitted with single organ dysfunction, with over 90% of patients requiring ≤ 3 days of respiratory support and over 70% requiring ≤ 3 days of vasopressor support. Only 3 patients were dialyzed during their stay, and of these, the longest was 3 days.

Reasons for DDH can be divided into three main groups: because the patient was medically fit and awaiting a ward bed (39.3%), because of a planned direct from ICU discharge (21.4%), or discharge against medical advice (DAMA) (39.3%). Where discharge location was specified, patients were discharged to their primary address (96.4%) or rehabilitation facilities (3.6%).

In total, 5 patients were readmitted within 1 year of discharge from the ICU, giving a readmission rate of 6%.

## Discussion

ICU practice is continuously evolving and is very unit-specific. The criteria for admission to ICU are constantly changing. They can frequently be affected by cultural values and resource constraints in the wider hospital, for example, lack of an HDU or unavailability of out-of-hours intermittent hemodialysis [4]. This leads to increased pressures for bed turnaround and a shift in practice where small numbers of patients are directly discharged from the ICU to home.

Our study aimed to evaluate the safety of this practice, as historically, it was uncommon and discouraged for patients to leave the ICU directly home rather than spending time in a step-down ward bed before discharge. Our hospital serves over 270,000 patients with just 26 dedicated intensive care beds, giving an ICU bed to population rate of 0.000096 or 1 bed for roughly 10,380 people. Given that the overall occupancy rate for hospital beds in Ireland is 93.8% [5], this explains the pressures both on ICU and ward beds and why this practice has evolved.

We must acknowledge some limitations. This is a monocentric study, and the generality of findings should be confirmed in a bigger cohort. Due to the limited sample size, we could not add more advanced statistical modeling to determine the factors associated with the patient’s characteristics. In addition, the pandemic might impact some of the patients’ type due to ICU admission constraints.

Acknowledging the practice of direct discharges will allow us to develop systems to follow up with these patients in the community. At present, our unit does not provide a post-ICU clinic. The first step may be introducing a system whereby patients are followed up by phone following discharge. It may also result in formalizing the pathway for direct discharges with primary teams to ensure follow-up tests and appointments are not missed Table 1.

**Table 1:** Patient characteristics

Patient characteristics	*N* (%)
**Total patients**	84
**Male**	56 (66.7)
**Female**	28 (33.3)
**Age (years)**	
**Age range**	20–81
**Admitted from**	
**Ed**	59 (70.3)
**Cath lab**	1 (1.2)
**CCU**	1 (1.2)
**Operating room**	14 (16.7)
**Transfer**	3 (3.6)
**Ward**	6 (7.1)
**Reason for admission**	
**Poisoning (accidental/deliberate)**	41 (49.2)
**Alcohol-related**	2 (2.4)
**Angioedema**	2 (2.4)
**Asthma**	2 (2.4)
**Pneumonia**	2 (2.4)
**Cardiogenic shock**	1 (1.2)
**Sepsis**	2 (2.4)
**COVID pneumonitis**	2 (2.4)
**Pulmonary embolism**	1(1.2)
**Emergency dialysis**	1(1.2)
**Hanging**	1(1.2)
**Head and neck/maxfax**	4 (4.8)
**Malignant hypertension**	1(1.2)
**Diabetic ketoacidosis**	3(3.6)
**Status epilepticus**	2 (2.4)
**Post-surgery (general/vascular)**	5(6)
**Post-cardiothoracic surgery**	1 (1.2)
**Rupture ul artery**	1 (1.2)
**Sickle cell crisis**	1 (1.2)
**SOL metastasis**	1 (1.2)
**Status epilepticus**	4 (4.8)
**Type 2 respiratory failure**	1 (1.2)
**UGIB hypovolemic shock**	2 (2.4)
**Out of hospital cardiac arrest**	1 (1.2)
**Length of stay (days)**	
**Range**	0–42
**Readmission rate**	5 (6)
**Supports (days)**	
**Vasopressors/ionotropes**	
**Mean**	1.72
**Respiratory**	
**Mean**	1.72
**Dialysis**	
**Mean**	0.08

Summary of patient characteristics, including primary diagnosis on admission, invasive supports required, and length of stay

## Conclusions

Although the practice of direct discharge from the ICU has been borne out of necessity due to an inadequate number of ICU and ward beds in our institution, it appears that in a select group of younger patients with minimal comorbidities and single organ failure, it may be safe to discharge them from the ICU to home directly.

## Data Availability

Not applicable.
